# #MedEd: Mapping the Current Landscape of Medical Education Discourse and Stakeholder Participation Across Social Media Platforms

**DOI:** 10.7759/cureus.39024

**Published:** 2023-05-15

**Authors:** Muhammad Hamza Shah, Sakshi Roy, Arjun Ahluwalia, Amer Harky

**Affiliations:** 1 School of Medicine, Dentistry and Biomedical Sciences, Queen's University Belfast, Belfast, GBR; 2 Cardiothoracic Surgery, Liverpool Heart and Chest Hospital, Liverpool, GBR

**Keywords:** teaching methodology, thematic analysis, continuous learning, medical education, social media

## Abstract

Background

Medical education is a constantly evolving and multifaceted field that requires ongoing discussion and innovation. Social media platforms have emerged as a popular medium for disseminating information and engaging in professional discourse among medical educators. In particular, the hashtag #MedEd has gained widespread recognition amongst individuals and organizations within the medical education community. Our objective is to gain insights into the types of information and discussions surrounding medical education, as well as the individuals or organizations involved in these conversations.

Methods

Searches were conducted across major social media platforms, including Twitter, Instagram, and Facebook, using the hashtag #MedEd. The top 20 posts posted on these platforms were analyzed through a reflexive thematic analysis approach utilizing the Braun and Clarke method. Furthermore, an examination was conducted on the profiles of those responsible for posting the aforementioned top posts, to ascertain the degree of participation from individuals versus organizations within the broader discourse pertaining to the topic.

Results

Our analysis revealed three thematic categories associated with the usage of the #MedEd hashtag, including discussions on "continuous learning and medical case presentations," "medical specialties and topics," and "medical education pedagogy." The analysis revealed that social media can serve as a valuable platform for medical education by providing access to a diverse range of learning resources, fostering collaboration and professional networking, and providing innovative teaching methods. Furthermore, profile analysis showed that individuals were more actively involved in the discussion of medical education topics on social media compared to organizations across all three platforms.

Conclusion

Our study highlights the significant role that social media platforms play in facilitating the exchange of information and ideas within the medical education community. The hashtag #MedEd serves as a means of connecting individuals and organizations across the globe, enabling them to engage in professional discourse and stay informed on the latest developments in the field. Our findings suggest that a better understanding of the thematic categories and stakeholders involved in medical education discussions on social media can aid educators, learners, and organizations in enhancing their engagement with this dynamic field.

## Introduction

Social media has increasingly become a popular medium for medical education due to its potential to enhance communication, collaboration, and knowledge dissemination [1,2]. Through social media, medical professionals can easily connect with colleagues, share resources, and participate in ongoing discussions and debates about important topics in the field. In addition, social media provides access to a wealth of educational materials, such as podcasts, videos, and webinars, which can supplement traditional forms of medical education [3].

Prior empirical research has shown that social media can be an effective tool for medical education. Cheston et al. carried out a systematic review on the use of social media in medical education and found that it can improve communication, facilitate collaboration, and enhance knowledge sharing among medical professionals [4]. In addition, social media has been shown to increase engagement and interest in medical education, particularly among younger generations of medical students and residents or trainees [5]. Furthermore, social media platforms also enable students to interact with educators regardless of their location, facilitating access to diverse perspectives and resources. Consequently, this allows for cross-cultural collaboration and knowledge sharing among professionals and students alike [4]. Not only this, but medical practitioners can also swiftly connect with colleagues from all over the world to share information and expertise, thus ensuring optimal care for patients under their purview as well as those under the care of other healthcare providers [6].

However, despite the potential benefits of social media for medical education, there are also concerns about the accuracy, reliability, and privacy of information shared on these platforms [7]. It is therefore important for medical professionals to critically evaluate the information they encounter on social media via established tools such as METRIQ-8 [8] and ensure that it is evidence-based and relevant to their clinical practice.

## Materials and methods

Data collection

The search was conducted between January 25, 2023, and February 25, 2023, and all posts that included the hashtag #MedEd during this period were considered for inclusion in the study. The search was limited to posts in English, as this is the primary language of medical education discourse on social media.

**Figure 1:**
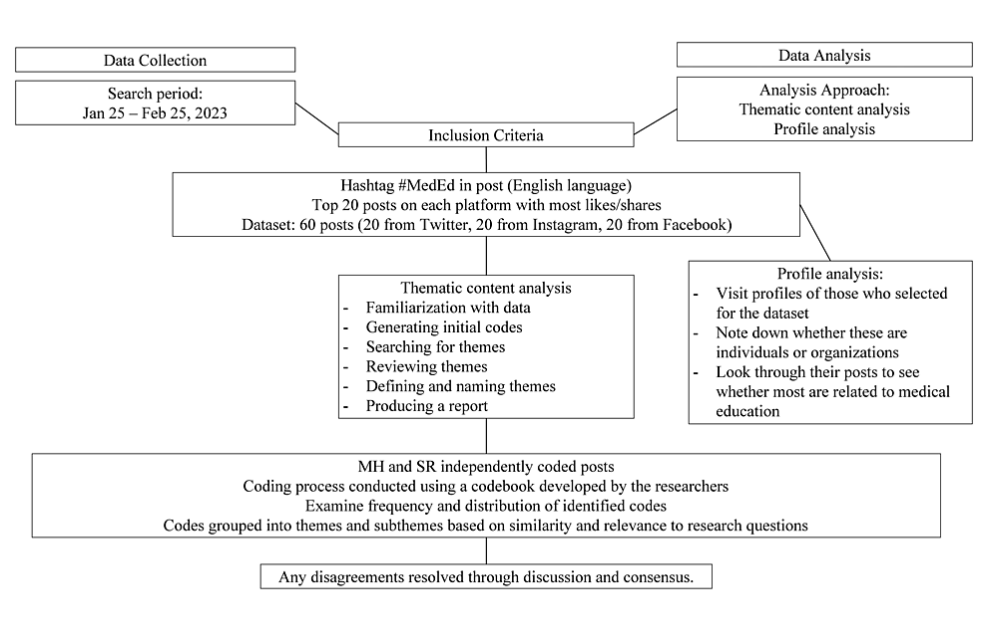
Flowchart depicting the data collection strategy

To ensure a representative sample of the discourse on medical education, we included the top 20 posts on each platform that received the highest number of likes and shares during the search period. These posts were selected as they were considered to be widely shared and hence would provide insights into pertinent themes in the discourse on medical education. Additionally, to avoid bias, the search was conducted independently by two researchers (MHS and SR) using the same search terms and criteria. Any discrepancies between the two searches were resolved through discussion and consensus. Our data collection strategy is summarized in Figure 1.

Thematic content analysis

A thematic content analysis approach was employed to analyze the top 20 posts on each platform. The analysis was guided by the Braun and Clarke method, which involves a series of methodical stages, namely, data familiarization, initial code generation, theme identification, theme review, theme definition or naming, and report production [9]. Two researchers independently coded the posts and then met to compare their coding and reconcile any discrepancies. Any disagreements were resolved through discussion and consensus. The coding process was conducted using a codebook developed by the researchers based on the research questions and the existing literature. After coding the data, we examined the frequency and distribution of the identified codes. The codes were then grouped into themes and subthemes based on their similarity and relevance to the research questions.

Profile analysis

Apart from analyzing the top 20 posts, we also conducted a thorough examination of the profiles of the individuals and organizations responsible for generating these posts; in this case, we have defined organizations as accounts managed by a company, non-profit organizations, or other entity for the purpose of promoting their brand and are active and engaging with MedTwitter. This was done to ascertain the level of involvement of each group in the discussion of medical education. Specifically, we recorded the number of posts related to medical education and the number of followers associated with each profile.

## Results

The thematic content analysis approach utilizing the Braun and Clarke method provided a structured and comprehensive way to analyze the top 20 posts on Twitter, Instagram, and Facebook linked with the hashtag #MedEd. In accordance with the selection of 20 high-yield posts across the three platforms, our final dataset consisted of 60 posts - 20 each from Twitter, Instagram, and Facebook - that were included in the analysis. The resulting thematic framework, presented in Table 1, reflects a comprehensive analysis of the data, highlighting the diversity and richness of the discussions related to medical education in the online sphere.

**Table 1:** Summary of the Braun and Clarke method steps applied in the analysis of social media content using the #MedEd hashtag

Steps in the Braun and Clarke method	Description	Example from #MedEd analysis
Step 1: Familiarization with the data	Researchers immerse themselves in the data to gain a general understanding of the content.	Two researchers independently read the top 20 tweets and posts on Twitter, Facebook, and Instagram using the #MedEd hashtag to get a sense of the overall content and identify initial ideas.
Step 2: Generating initial codes	Researchers generate initial codes to identify patterns and concepts within the data.	Initial codes such as "topics," "curriculum," "diagnosis," and "assessment" were identified by both researchers and recorded in a codebook.
Step 3: Searching for themes	Researchers search for potential themes by grouping codes that share similar concepts or patterns.	Both researchers independently searched for themes by organizing the initial codes into potential groupings, which were then compared and refined.
Step 4: Reviewing themes	Researchers review and refine potential themes to ensure that they accurately capture the content of the data.	The researchers reviewed the identified themes, refined them, and organized them into a hierarchical structure to show their relationship to each other.
Step 5: Defining and naming themes	Researchers define and name each theme to accurately capture its essence.	The themes were defined and named, such as "continuous learning," "medical specialties," and "assessment and evaluation of learners."
Step 6: Producing the report	Researchers produce a final report that includes the identified themes and supporting data.	The final report includes the identified themes, examples of supporting data, and a discussion of their relevance to medical education.

Theme 1: “Continuous learning and medical case presentations”

This theme is centered around the provision of valuable learning resources and case studies through tweets and posts on social media platforms, which can aid medical students and professionals in keeping up-to-date with the latest medical information and best practices.

**Figure 2:**
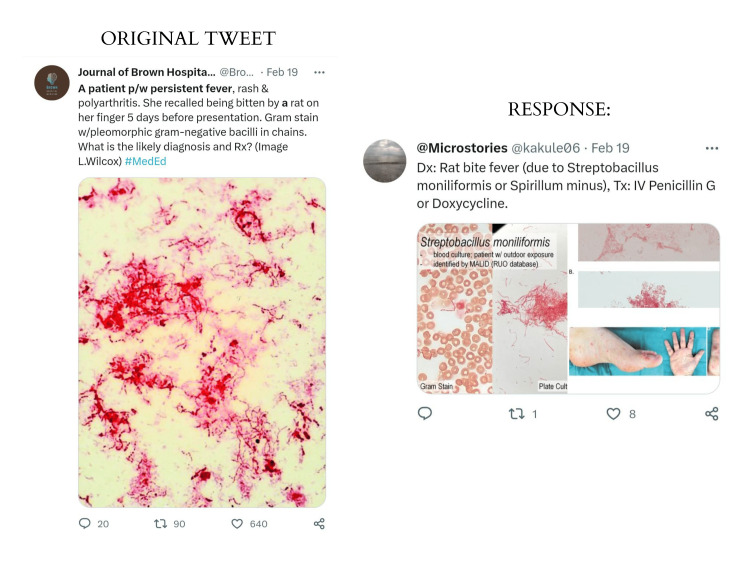
A medical case tweet with the relevant hashtag and response

The tweets and posts under this theme often showcase unique and novel medical case presentations that may not be encountered in daily medical practice. Figure 2 depicts an example of the kind of valuable learning resources that may be useful to both educators and learners.

Additionally, Table 2 outlines the learning potential of the tweet as a medical education resource using a structured approach.

**Table 2:** Learning potential of the tweet as a medical education resource

Key features: This is a tweet that presents a medical case, including a histological image, and asks the audience to provide a diagnosis or contribute to the discussion on possible treatment plans.
Potential audience: Medical professionals, students, and educators interested in learning about medical cases and diagnostic techniques.
Benefits: By asking for a diagnosis or contribution to the discussion, this tweet promotes critical thinking and problem-solving skills among medical professionals. The hashtag #MedEd used in the tweet can provide opportunities for medical professionals to engage in continuing medical education (CME) and foster discussion and collaboration among medical professionals.
Challenges: Ensuring patient privacy and confidentiality and avoiding inappropriate or unprofessional commentary or diagnoses.
Best practice: Obtain appropriate consent from patients before sharing their cases. Ensure that all commentary and diagnoses are respectful, evidence-based, and appropriate for the intended audience. Encourage discussion and collaboration among participants.
Responses: The responses to the tweet include a range of possible answers for diagnosis and treatment, as well as histological and patient images for differing presentations.

Both MHS and SR devised this structure after considering which parameters would be particularly relevant to themselves as learners in the preliminary stages of their medical careers.

Theme 2: “Medical specialties and topics”

The theme elucidates the broad spectrum of medical specialties and conditions that attract the attention of medical students and professionals. The posts falling under this theme furnish valuable information and perspectives concerning various medical conditions and treatments. Their aim is to equip medical students with essential knowledge regarding the presentation, diagnosis, and treatment of such conditions, thereby assisting them in preparing for clinical practice. Accordingly, these posts can prove to be an invaluable resource for medical students.

**Figure 3:**
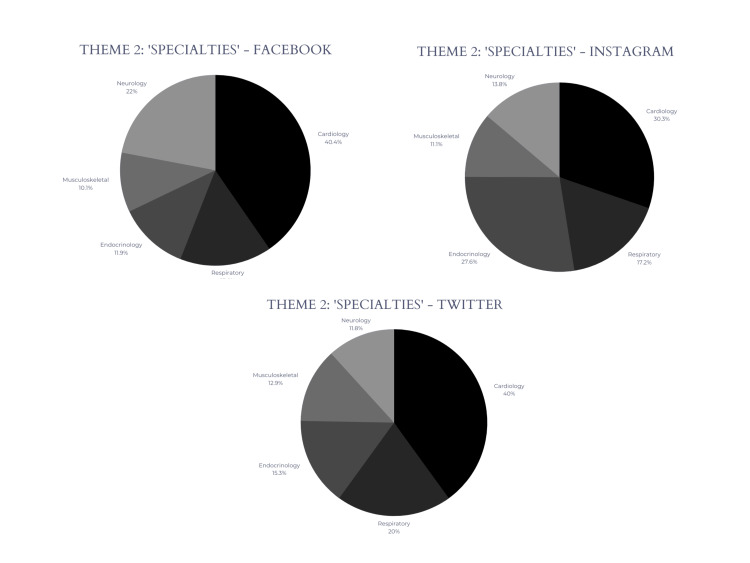
Distribution of medical specialties across social media platforms

To visually depict the distribution of each specialty across the three social media platforms, pie charts have been presented in Figure 3 below.

Theme 3: “Pedagogical and teaching strategies”

The theme underscores the significance of pedagogical and teaching strategies in medical education. The posts under this theme emphasized the essentiality of employing effective teaching methods that can facilitate the learning process of medical students. Overall, they ranged from discussions on different teaching approaches to the use of technology and its impact on medical education.

The posts encapsulated in this theme provide valuable insights into the diverse approaches and methods that are currently being employed in medical education. This information can aid medical educators in integrating innovative teaching techniques and strategies in their classrooms to augment the learning experience of their students.

Profile analysis

The results of the profile analysis across all three social media platforms revealed that the majority of the top posts were made by individuals rather than healthcare organizations. On Facebook, 70% of the top posts were made by individuals, while 30% were made by organizations. Similarly, on Twitter, 65% of the top posts were made by individuals, while 35% were made by organizations. Interestingly, on Instagram, 85% of all top posts were made by individuals, while only 15% were made by organizations.

**Figure 4:**
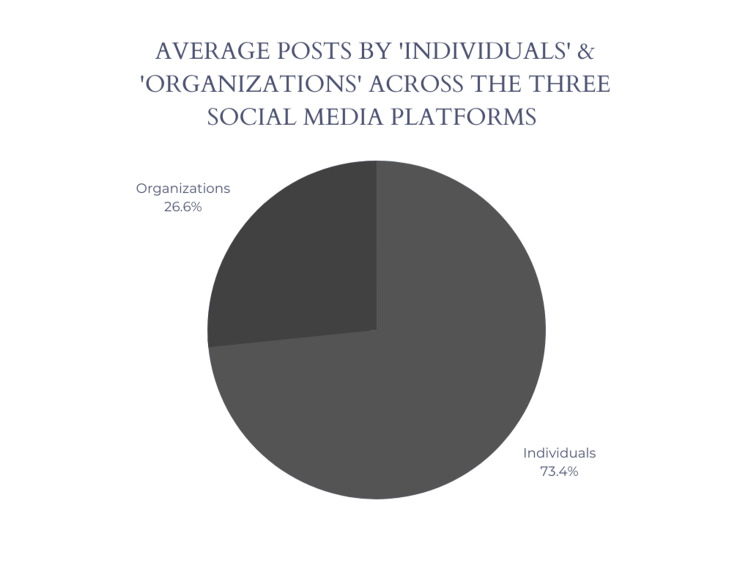
Average percentage of posts by individuals and organizations across the three social media platforms

In terms of the number of posts related to medical education, the profiles of individuals had a wider range of posts, with some having hundreds or even thousands of posts related to medical education, while others had only a few. On the other hand, the profiles of organizations had a more consistent number of posts related to medical education, with most having between 50 and 100 posts and 100+ tweets. Figure 4 showcases the average for both individuals and organizations across the three platforms.

The results of the profile analysis indicate that there is a significant presence of individual users in the discussion of medical education on social media, with individuals being the dominant contributors to the top posts across all three platforms. This confirms that social media equally provides a platform for individual users such as medical professionals or medical students to engage in discussions and share their perspectives on medical education. The higher percentage of organizational posts on Facebook and Instagram compared to Twitter suggests that organizations may be more active in sharing their content and engaging with their followers on these platforms. On the other hand, the majority of top posts being made by individuals on Twitter may indicate a greater emphasis on personal opinions and experiences.

## Discussion

This study explored the landscape of medical education on social media platforms by analyzing the types of information and discussions surrounding medical education, as well as the individuals or organizations involved in these conversations. The analysis revealed a range of thematic categories associated with the usage of the #MedEd hashtag, including discussions on medical education pedagogy, medical specialties, and continuous medical education. Furthermore, our profile analysis indicated that key players within these conversations are medical educators, learners, and professional organizations, with individuals making up 73.4% of the top 20 posts across the three social media platforms.

One of the major advantages of this study is that it provides a comprehensive overview of the #MedEd hashtag on social media, which has not been explored in depth before. By analyzing the top 20 posts on Twitter, Instagram, and Facebook, the study captures the most significant themes and trends in the discourse on medical education, which can be represented in the literal sense via this hashtag. Moreover, the investigation employed the Braun and Clarke methodology of thematic content analysis, a rigorous and widely acknowledged approach for analyzing qualitative data, provided that it is appropriately applied [10]. The utilization of two independent researchers at each stage of the analysis process contributes significantly to the robustness of the methodology, as it enhances the reliability and validity of derived themes. By adhering to this systematic approach, the content analysis process was able to identify and develop relevant themes that accurately captured the essence of the qualitative data, thereby adding a further layer of rigor to the entire analytical procedure. Another advantage of this study is that it sheds light on the extent of individual versus organizational involvement in the discussion of medical education on social media. The profile analysis showed that both individuals and organizations are actively involved in these conversations. This is important because it highlights the potential for collaboration and partnerships between different stakeholders in the medical education community.

The findings of this study have several implications for medical educators, learners, and professional organizations. Firstly, the study highlights the importance of social media in facilitating the exchange of information and ideas within the medical education community. The hashtag #MedEd serves as a means of connecting individuals and organizations across the globe, enabling them to engage in professional discourse and stay informed on the latest developments in the field. Not only this but the case presentations mentioned theme one can serve as an excellent learning opportunity for medical students and professionals, allowing them to expand their knowledge and understanding of complex medical conditions [11]. Secondly, the study provides insights into the thematic categories and stakeholders involved in medical education discussions on social media, which can aid educators, learners, and organizations in enhancing their engagement with this dynamic field. These ideas are consistent with social-constructivist principles proposed by Dewey and Vygotsky, which suggest that social interaction, sharing of information, and active participation in social media activities may facilitate learning [12]. Additionally, since effective teaching methods play a pivotal role in preparing medical students to become skilled and competent healthcare professionals [13], it is crucial for educators to adapt their teaching strategies to keep pace with the constantly evolving nature of the medical field. Therefore, theme 3 builds upon why it is imperative for educators to incorporate teaching strategies that can be tailored to adapt to these changes.

However, it is important to acknowledge the limitations of this study. Firstly, the study was limited to posts in English, which may not capture the full spectrum of medical education discourse on social media. Secondly, the study only analyzed the top 20 posts on each platform, which is not representative of the entire discourse on medical education. Thirdly, the study did not evaluate the accuracy, reliability, and privacy of information shared on social media, which are important concerns in the medical education community.

Here it is important to note that while our study focused on the use of the #MedEd hashtag on social media platforms, it is important to note that social media is just one of many tools available for medical education. Traditional forms of medical education, such as lectures and textbooks, remain important sources of information for learners [14]. However, social media offers unique advantages in terms of accessibility, interactivity, and reach [6,15]. As such, medical educators and learners should consider integrating social media into their educational strategies to supplement and enhance traditional forms of medical education.

## Conclusions

The present study provides valuable insights into the landscape of medical education on social media platforms, demonstrating that social media is a powerful tool for medical education. Our findings highlight the potential of social media to connect individuals and organizations, enabling them to engage in discussions and debates about important topics in the field. This deeper understanding of the types of information and discussions surrounding medical education on social media can help stakeholders enhance their engagement with this dynamic field. For medical educators, social media offers a valuable platform for sharing educational resources, collaborating with colleagues, and staying up-to-date on the latest developments in the field. For learners, social media can provide access to a wealth of educational materials and opportunities to engage in discussions with their peers and mentors. Moreover, for professional organizations, social media can facilitate communication and collaboration with members, as well as provide opportunities for advocacy and outreach.

## References

[1] Grajales FJ 3rd, Sheps S, Ho K, Novak-Lauscher H, Eysenbach G (2014). Social media: a review and tutorial of applications in medicine and health care. J Med Internet Res.

[2] D'souza F, Shah S, Oki O, Scrivens L, Guckian J (2021). Social media: medical education's double-edged sword. Future Healthc J.

[3] Galiatsatos P, Porto-Carreiro F, Hayashi J, Zakaria S, Christmas C (2016). The use of social media to supplement resident medical education - the SMART-ME initiative. Med Educ Online.

[4] Cheston CC, Flickinger TE, Chisolm MS (2013). Social media use in medical education: a systematic review. Acad Med.

[5] Wang CX, Kale N, Miskimin C, Mulcahey MK (2021). Social media as a tool for engaging medical students interested in orthopaedic surgery. Orthop Rev (Pavia).

[6] Ventola CL (2014). Social media and health care professionals: benefits, risks, and best practices. P T.

[7] Vukušić Rukavina T, Viskić J, Machala Poplašen L, Relić D, Marelić M, Jokic D, Sedak K (2021). Dangers and benefits of social media on e-professionalism of health care professionals: scoping review. J Med Internet Res.

[8] Colmers-Gray IN, Krishnan K, Chan TM (2019). The revised METRIQ score: a quality evaluation tool for online educational resources. AEM Educ Train.

[9] Braun V, Clarke V (2006). Using thematic analysis in psychology. Qual Res Psychol.

[10] Braun V, Clarke V (2014). What can "thematic analysis" offer health and wellbeing researchers?. Int J Qual Stud Health Well-being.

[11] Pershad Y, Hangge PT, Albadawi H, Oklu R (2018). Social medicine: Twitter in healthcare. J Clin Med.

[12] Kirch SA, Sadofsky MJ (2021). Medical education from a theory-practice-philosophy perspective. Acad Pathol.

[13] Burgess A, van Diggele C, Roberts C, Mellis C (2020). Key tips for teaching in the clinical setting. BMC Med Educ.

[14] AlQhtani A, AlSwedan N, Almulhim A (2021). Online versus classroom teaching for medical students during COVID-19: measuring effectiveness and satisfaction. BMC Med Educ.

[15] Guckian J, Utukuri M, Asif A (2021). Social media in undergraduate medical education: a systematic review. Med Educ.

